# A Case Report of Milk-Alkali Syndrome Secondary to Excessive Antacid Use

**DOI:** 10.5811/cpcem.48354

**Published:** 2026-01-01

**Authors:** Samir Beso, Layla Abubshait

**Affiliations:** * Jefferson Einstein Montgomery Hospital, Department of Emergency Medicine, East Norriton, Pennsylvania; † Sidney Kimmel Medical College, Thomas Jefferson University, Philadelphia, Pennsylvania

**Keywords:** milk-alkali syndrome, hypercalcemia, metabolic alkalosis, acute kidney injury, antacids, case report

## Abstract

**Introduction:**

Milk-alkali syndrome is characterized by the triad of hypercalcemia, metabolic alkalosis, and acute kidney injury resulting from excessive intake of calcium and absorbable alkali. Despite falling out of prominence with the advent of modern ulcer treatments, milk-alkali syndrome has experienced a resurgence with the widespread availability of over-the-counter calcium preparations, which now account for up to 10% of hypercalcemia cases.

**Case Report:**

A 60-year-old man with multiple comorbidities presented to the emergency department with altered mental status after his scheduled kyphoplasty was canceled due to concerning neurological findings. Laboratory evaluation revealed severe hypercalcemia, marked metabolic alkalosis, and acute kidney injury. Further history revealed excessive antacid consumption for heartburn. The patient was diagnosed with milk-alkali syndrome, treated with intravenous fluids and calcitonin, and discharged home after 48 hours with complete resolution of signs and symptoms.

**Conclusion:**

Milk-alkali syndrome represents an increasingly recognized cause of severe hypercalcemia in the emergency setting. This case demonstrates the importance of thorough medication history, early recognition of the classic triad, and prompt initiation of conservative management. With the growing use of calcium-based, over-the-counter preparations, emergency physicians must maintain vigilance for this potentially serious but readily treatable condition.

## INTRODUCTION

Milk-alkali syndrome is characterized by the triad of hypercalcemia, metabolic alkalosis, and acute kidney injury resulting from excessive intake of calcium and absorbable alkali. Although historically associated with milk and bicarbonate treatment for peptic ulcer disease, modern cases typically involve overconsumption of calcium-containing antacids or supplements.^1^ Despite falling out of prominence with the advent of modern ulcer treatments, milk-alkali syndrome has seen a resurgence with the widespread availability of over-the-counter (OTC) calcium preparations. It accounts for up to 10% of hypercalcemia cases and represents the third most common cause of hospital-associated hypercalcemia after hyperparathyroidism and malignancy.^2^ The diagnosis can be easily missed, leading to unnecessary investigations and inappropriate treatment.

## CASE REPORT

A 60-year-old man with type two diabetes on metformin, hyperlipidemia on rosuvastatin, hypertension on metoprolol, and prior myocardial infarction presented to the emergency department after his scheduled first lumbar vertebrae kyphoplasty was cancelled due to altered mental status noted by the interventional radiology team. The patient reported feeling progressively disoriented over five days, accompanied by significant heartburn. Initially, he did not volunteer information about his antacid use. However, when the emergency physician specifically inquired about OTC medications and treatments for his heartburn symptoms, the patient revealed he had been self-medicating with calcium carbonate tablets, consuming more than 20 tablets daily during this period.

Vital signs revealed the following: heart rate, 110 beats per minute; blood pressure, 138/82 millimeters of mercury; temperature, 98.6 °F; respiratory rate, 18 breaths per minute; and oxygen saturation, 98% on room air. The patient appeared tired but remained alert and oriented. Physical examination was otherwise unremarkable with no focal neurological deficits. Computed tomography angiography of the head and neck was performed due to the acute onset of neurological symptoms and concern for cerebrovascular accident, which was negative for acute cerebrovascular pathology.

Laboratory studies and electrocardiogram were ordered, revealing multiple electrolyte abnormalities and prolonged corrected QT interval (Image) (Table).

The combination of severe hypercalcemia, metabolic alkalosis, and acute kidney injury suggested milk-alkali syndrome given the history of excessive antacid use, although thiazide diuretic use, primary hyperparathyroidism, and malignancy-associated hypercalcemia were also considered. Given the constellation of severe hypercalcemia, metabolic alkalosis, and the patient’s history of excessive antacid consumption, milk-alkali syndrome was strongly suspected. The emergency team initiated aggressive intravenous (IV) fluid resuscitation with 0.9% normal saline at 200 milliters per hour to promote calciuresis and correct volume depletion. To achieve more rapid calcium reduction, subcutaneous calcitonin 4 units/kilogram was administered. While furosemide was considered for its calciuric effects, it was initially deferred due to concerns about further volume depletion in this already dehydrated patient. Given the severity of his hypercalcemia and altered mental status, the patient was admitted to the internal medicine service for continued monitoring and management.

*CPC-EM Capsule*

What do we already know about this clinical entity?

*Milk-alkali syndrome has resurged with widespread use of over-the-counter calcium preparations for heartburn.*

What makes this presentation of disease reportable?

*This case illustrates the challenge of diagnosis when patients don’t report antacid use, and the paradoxical electrocardiogram findings that can occur with concurrent metabolic derangements.*

What is the major learning point?

*Emergency physicians must specifically inquire about over-the-counter antacid consumption.*

How might this improve emergency medicine practice?

*Proactive medication reconciliation including specific questions about heartburn treatments can lead to faster recognition of milk-alkali syndrome.*

The patient’s clinical response was reassuring and consistent with milk-alkali syndrome. Continued IV. hydration throughout hospital day (HD) 1 resulted in gradual improvement of his calcium levels to 12.1 milligrams per deciliter (mg/dL), However, recognizing that calcium remained significantly elevated despite aggressive hydration, the medical team administered furosemide 40 mg IV on HD 2 to enhance calcium excretion, with repeat calcium levels normalizing to 8.8 mg/dL. Concurrent with calcium normalization, the patient’s sodium levels improved to 134 milliequivalents/L as volume status was restored.

During his hospitalization, the patient experienced persistent cognitive signs and symptoms including speech slurring, which prompted concern for possible cerebrovascular pathology. Magnetic resonance imaging of the brain without contrast was obtained, revealing an incidental subacute infarct in the right frontal lobe. Neurology consultation was obtained, and the patient was prescribed apixaban 5 mg by mouth twice daily for secondary stroke prevention. Notably, his mental status improvements paralleled the correction of his hypercalcemia, suggesting that the initial altered mental status was primarily attributable to the severe calcium elevation rather than the stroke itself.

Endocrinology consultation was also obtained to help guide further management and evaluate for underlying metabolic disorders. The endocrinology team confirmed that the patient’s normal-range parathyroid hormone levels were appropriate given the severe hypercalcemia, and they noted significant vitamin D deficiency, which likely contributed to the clinical picture. They recommended outpatient follow-up for thyroid ultrasound due to subclinical hypothyroidism, evidenced by low thyroid-stimulating hormone in the setting of normal triiodothyronine and thyroxine levels. The patient was discharged on HD 2 with instructions to avoid calcium-containing antacids and follow up with his primary care physician and newly arranged endocrinology consultation.

## DISCUSSION

This case exemplifies the classic presentation of milk-alkali syndrome in the modern era. The syndrome’s resurgence correlates with increased OTC calcium carbonate use, particularly among patients self-treating gastrointestinal (GI) symptoms.^4^ Recent literature continues to document cases of milk-alkali syndrome, emphasizing its ongoing clinical relevance despite advances in peptic ulcer disease management.^5^,^6^

The syndrome results from complex interactions between excessive calcium and alkali intake, leading to impaired renal excretion of both substances. Volume depletion, which can occur from various mechanisms including reduced oral intake or GI losses, further exacerbates the condition by reducing glomerular filtration and enhancing calcium reabsorption. The pathophysiology involves a vicious cycle whereby hypercalcemia leads to nephrogenic diabetes insipidus and volume depletion, which in turn worsens calcium retention.

Clinical recognition relies on identifying key diagnostic features including a history of excessive calcium-containing antacid use, and the classic triad of hypercalcemia, metabolic alkalosis, and acute kidney injury, along with appropriately suppressed or normal parathyroid hormone levels. The rapid clinical improvement with conservative management serves as both a diagnostic and therapeutic confirmation. Physicians must maintain proactive medication reconciliation practices, as patients often do not spontaneously report OTC antacid use, viewing them as harmless supplements rather than medications with potential for toxicity.

Emergency management centers on immediate discontinuation of calcium-containing products as the most crucial intervention. Aggressive IV hydration remains the cornerstone of treatment, promoting calciuresis and correcting the volume depletion that perpetuates the syndrome. Calcitonin provides rapid but temporary calcium reduction, typically within four to six hours, making it useful for severe cases requiring immediate intervention. Loop diuretics should be used cautiously and only after adequate volume resuscitation to avoid worsening dehydration. Bisphosphonates are generally unnecessary given the rapid response to conservative measures, unlike other causes of severe hypercalcemia.

The paradoxical corrected QT interval prolongation observed in this case, typically shortened in hypercalcemia, likely resulted from concurrent metabolic derangements including severe hyperglycemia and ketosis. This finding illustrates the complex interplay of multiple metabolic abnormalities that can occur in severely ill patients. The incidental stroke finding, while unrelated to milk-alkali syndrome, emphasizes the importance of comprehensive evaluation in elderly patients with altered mental status, as multiple pathological processes may coexist.

Patient education regarding appropriate antacid use is crucial for preventing recurrence. Patients should be counseled on recommended dosing limits for OTC calcium preparations and advised to seek medical attention for persistent GI symptoms rather than escalating self-treatment. Physicians should consider concurrent medical conditions that may complicate the clinical presentation and require additional management, as demonstrated by this patient’s diabetic ketoacidosis and cerebrovascular disease.

## CONCLUSION

Milk-alkali syndrome represents an increasingly recognized cause of severe hypercalcemia in the emergency setting. This case demonstrates the importance of thorough medication history, early recognition of the classic triad, and prompt initiation of conservative management. With the growing use of calcium-based over-the-counter preparations, emergency physicians must maintain vigilance for this potentially serious but readily treatable condition.

## Figures and Tables

**Image:**
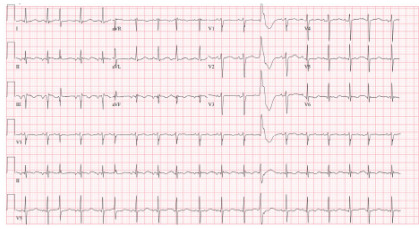
Electrocardiogram showed sinus tachycardia at a rate of 110 beats per minute with QTc (corrected QT interval) prolongation (512 milliseconds). Notable findings include normal axis, normal QRS complex width, and no evidence of ischemic changes. The corrected QT interval prolongation is paradoxical given the severe hypercalcemia, which typically causes corrected QT interval shortening.

**Table:** Laboratory results during emergency department evaluation and hospitalization of patient with severe hypercalcemia. metabolic alkalosis, and acute kidney injury associated with excessive antacid consumption.

Test	ED Results	Hospital Day 1	Hospital Day 2	Normal Range
Emergency Department Labs				
Sodium	129 mEq/L			136–146 mEq/L
Potassium	4.0 mEq/L			3.6–5.1 mEq/L
Chloride	73 mEq/L			98–107 mEq/L
BUN	52 mg/dL			8–26 mg/dL
Creatinine	1.27 mg/dL			0.70–1.20 mg/dL
Calcium	15.8 mg/dL	12.1 mg/dL	8.8 mg/dL	8.4–10.3 mg/dL
Glucose	485 mg/dL			70–99 mg/dL
**Inpatient Labs**				
Parathyroid Hormone		20.8 pg/mL		9.0–73.0 pg/mL
25-Hydroxy Vitamin D		7 ng/mL		30–100 ng/mL
TSH		0.3 mcIU/mL		0.35–4.94 mcIU/mL
Free T4		1.21 ng/dL		0.70–1.48 ng/dL

*BUN*, blood urea nitrogen; *ED*, emergency department; *mEq/L*, milliequivalents per liter; *mg/dL*, milligrams per deciliter; *mcIU/mL*, microinternational units per milliliter; *ng/mL*, nanograms per milliliter; *pg/mL*, picograms per milliliter; *TSH*, thyroid stimulating hormone; *T4*, thyroxine.
